# Molecular Modeling of Peroxidase and Polyphenol Oxidase: Substrate Specificity and Active Site Comparison

**DOI:** 10.3390/ijms11093266

**Published:** 2010-09-14

**Authors:** Prontipa Nokthai, Vannajan Sanghiran Lee, Lalida Shank

**Affiliations:** 1 Bioinformatics Research Laboratory (BiRL), Faculty of Science, Chiang Mai University, Chiang Mai 50200, Thailand; E-Mail: pnokthai@gmail.com; 2 Computational Simulation and Modeling Laboratory (CSML), Faculty of Science, Chiang Mai University, Chiang Mai 50200, Thailand; 3 Department of Chemistry and Center for Innovation in Chemistry, Faculty of Science, Chiang Mai University, Chiang Mai 50200, Thailand; 4 Thailand Center of Excellence in Physics (ThEP), Commission Higher on Education, Ministry of Education, Bangkok 10400, Thailand; 5 Phytochemica Research Unit, Faculty of Science, Chiang Mai University, Chiang Mai 50200, Thailand

**Keywords:** peroxidase, polyphenol oxidase, browning reaction, molecular docking

## Abstract

Peroxidases (POD) and polyphenol oxidase (PPO) are enzymes that are well known to be involved in the enzymatic browning reaction of fruits and vegetables with different catalytic mechanisms. Both enzymes have some common substrates, but each also has its specific substrates. In our computational study, the amino acid sequence of grape peroxidase (ABX) was used for the construction of models employing homology modeling method based on the X-ray structure of cytosolic ascorbate peroxidase from pea (PDB ID:1APX), whereas the model of grape polyphenol oxidase was obtained directly from the available X-ray structure (PDB ID:2P3X). Molecular docking of common substrates of these two enzymes was subsequently studied. It was found that epicatechin and catechin exhibited high affinity with both enzymes, even though POD and PPO have different binding pockets regarding the size and the key amino acids involved in binding. Predicted binding modes of substrates with both enzymes were also compared. The calculated docking interaction energy of trihydroxybenzoic acid related compounds shows high affinity, suggesting specificity and potential use as common inhibitor to grape ascorbate peroxidase and polyphenol oxidase.

## 1. Introduction

Browning of vegetables, fruits and flowers alter their appearances, flavors, textures, and lower their marketing values. Appearance, which is significantly impacted by color, is one of the first attributes used by consumers to evaluate the quality of goods [1]. The browning process can be caused by both enzymatic and non-enzymatic biochemical reactions [2]. Polyphenol oxidase (PPO) and peroxidase (POD) are two well known enzymes involved in the browning process [1–3]. PPO catalyzes the conversion of phenolic compounds to quinones and assists their products’ polymerization. Its catalysis, in the presence of oxygen, leads to the formation of undesirable brown pigments and off-flavored products [4]. The browning of injured, peeled or diseased fruit tissues can causes undesirable quality changes during handling, processing and storage.

PPO is a dicopper-containing enzyme. Several studies have reported the involvement of PPO in the oxidation of the polyphenols from plants. PPO activity can be monitored by oxygen consumption or spectrophotometrically using a variety of substrates such as pyrogallol, pryocatechol, 4-methylcatechol, 3,4-dihydroxyphenylacetic acid, 4-tert-butylcatechol and chlorogenic acid [5]. PPO shows high activity with diphenols [6]. Two kinds of reactions generated by PPO are the hydroxylation of monophenols to *o*-diphenol and the oxidation of *o*-diphenol to *o*-quinone [3]. The schematic reaction catalyzed by PPO is as follows:

$$\text{monophenol}\overset{\text{PPO}+{\text{O}}_{2}}{\to }\text{diphenol}\overset{\text{PPO}+{\text{O}}_{2}}{\to }\underset{\begin{array}{c} ↓\\ \text{complex brown polymer} \end{array}}{o-\text{quinone}}$$

POD can be found in plants, animals and microbes. It is one of the most thermostable enzymes responsible for performing single electron oxidation on a wide variety of compounds, in the presence of hydrogen peroxide. POD reduces H_2_O_2_ to water while oxidizing a variety of substrates. The catalytic process of POD occurs in a multistep reaction. This is shown in the following scheme [7]:

$$\begin{array}{l} \text{POD}+{\text{H}}_{2}{\text{O}}_{2}\to \text{Compound I}+{\text{H}}_{2}\text{O}\\ \text{Compound I}+\text{S}\to \text{Compound II}+{\text{S}}^{•}\\ \text{Compound II}+\text{S}\to \text{POD}+{\text{S}}^{•} \end{array}$$

S stands for substrate and S^•^ represents the corresponding radical. AH_2_ and AH^•^ represent a reducing substrate and its radical product, respectively. A simplified equation for this chemical reaction is as follows:

$${\text{H}}_{2}{\text{O}}_{2}+2{\text{AH}}_{2}\overset{\text{POD}}{\to }2{\text{H}}_{2}\text{O}+2{\text{AH}}^{•}$$

An interdependence between prevention of off-flavor development and inactivation of POD enzyme in frozen vegetables has been reported [4]. Furthermore, the related activity of PPO and POD is due to the generation of hydrogen peroxide during the oxidation of phenolic compounds in PPO-catalyzed reactions [3,8,9].

The catalytic reactions of the oxidative enzymes, POD and PPO, have been studied in fruits and vegetables for many years. Both enzymes have some common substrates, but each also has its specific substrates [5,6,10,11]. Their common diphenolic substrates lead to products with brown colors. Moreover, both enzymes have some common inhibitors and some specific inhibitors. Inhibition of POD and PPO activities can reduce the browning process. A binding of ligand and protein may result in the activation or the inhibition of the enzyme [11,12]. The aim of this study was to investigate the binding pattern of substrates and inhibitors of PPO and POD of grape, *Vitis vinifera*, using a computational method. Three–dimensional models of grape POD and PPO were constructed, and substrate specificity, binding site of enzymes, and the activity of the selected substrates and inhibitors were compared using the molecular docking. A comparison of theoretical and experimental results of enzyme activity was also investigated.

## 2. Results and Discussion

### 2.1. Three-Dimensional Model of Grape Ascorbate Peroxidase and Polyphenol Oxidase

**Figure 1:**
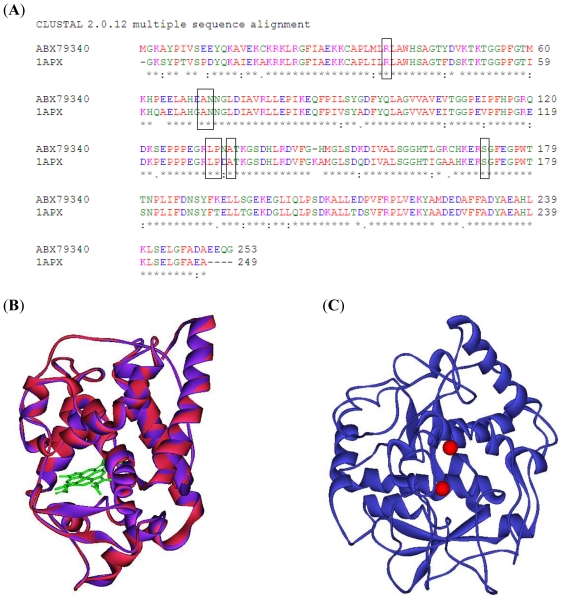
(**A**) Sequence alignment of ABX79340 and 1APX produced by ClustalW2. The residues in blocks are the amino acids in the binding site; (**B**) Superimposition of the grape peroxidase homology model (violet) and pea cytosolic ascorbate peroxidase (red). Heme molecule is shown as a green stick; (**C**) X-ray structure of grape polyphenol oxidase (2P3X). Red circles are copper atoms.

**Figure 2:**
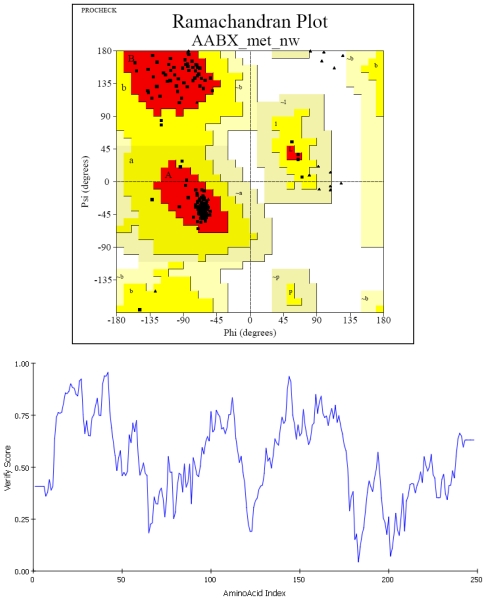
Ramachandran plot (top) of the Psi/Phi distribution of the grape ascorbate peroxidase homology model produced by PROCHECK and the structure evaluation with Profiles-3D (bottom). The favored and most favored regions are yellow and red, respectively. Pale yellow is the generally allowed region and disallowed region is white.

By BLAST searching, five structurally determined peroxidases, including cytosolic ascorbate peroxidase of pea (1APX; 80% identity), cytosolic ascorbate peroxidase of soybean (2GHC; 79% identity), chloroplastic ascorbate peroxidase of tobacco (1IYN; 42% identity), cytochrome c peroxidase of yeast (2EUT; 34% identity) and peroxidase of Arabidopsis (1PA2; 32% identity) were found with substantial sequence similarity to that of grape peroxidase. The first three peroxidases share similar structural fold and can be used as structural template in the homology modeling of grape peroxidase. Conserved residues in the binding pocket among the selected peroxidase are Arg, Ala, Asn, Leu, Pro, Ala and Ser, as highlighted in the box in Figure 1. As a result, 1APX—with the highest sequence similarity to grape peroxidase (80% sequence identity and an E-value of 2.62E-116)—was chosen as the template. A sequence alignment of grape ascorbate peroxidase (ABX79340) and 1APX was produced by ClustalW2.0 [13] with default parameters as shown in Figure 1A. A superimposed image of the grape peroxidase homology model and the pea cytosolic ascorbate peroxidase model is shown in Figure 1B. After structure refinement, the quality of the residue backbone conformations in the grape peroxidase model was checked by PROCHECK (http://www.jcsg.org/scripts/prod/validation/sv_final.cgi) as shown by Ramachandran Plot (Figure 2). In the diagram, the white areas correspond to conformations where atoms in the polypeptide come closer than the sum of their van der Waals radii. These regions are sterically disallowed for all amino acids except glycine (as shown by triangles), which is unique in that it lacks a side chain. The red and yellow regions correspond to conformations of the allowed regions, namely the beta-sheet and alpha-helical conformations. There are 95.6% residues in the most favored regions and 4.4% of residues in additional allowed regions (Table 1) and the overall G-factor is 0.12 Å. Assessing for compatibility of each residue of minimized Model A was checked by Verify 3D analysis in Discovery Studio. The verified score of an amino acid residue indicates that a low score is given to a hydrophobic residue on a protein’s surface and a polar residue in the protein’s core. Regions of the protein for which the score approaches zero or become negative are likely to be misfolded. In Figure 2 (bottom), some amino acids (Ile183, Ile201) are in the poor region; however, they are located outside and far away from the binding pocket.

### 2.2. Comparison of Substrate Binding Site for PPO and POD from Molecular Docking

**Figure 3:**
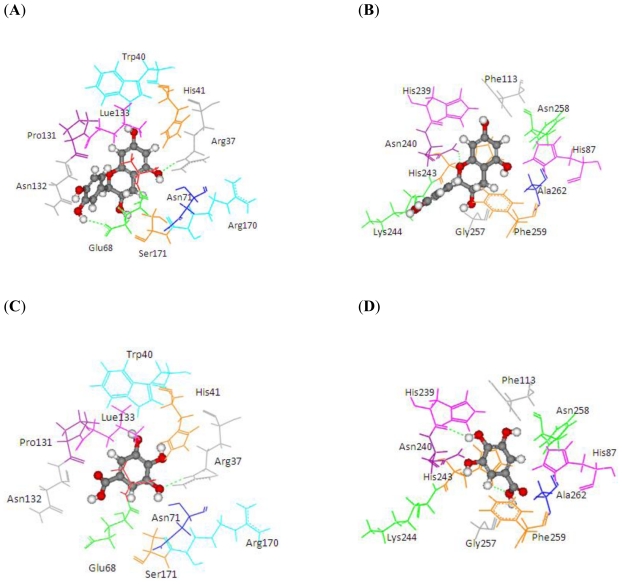
3 Å binding site comparison of PPO and POD with common substrate and inhibitor (in ball and stick model). Dashed line represents H-bond. (**A**) POD with EPC; (**B**) PPO with EPC; (**C**) POD with 3,4,5-THBA; (**D**) PPO with 3,4,5-THBA.

The docking of grape peroxidase and polyphenol oxidase with each substrate, 4-methylcatechol, guaiacol, pyrogallol, 3,4-dihydroxyphenyl acetic acid, catechin and epicatechin, were calculated. The conformation with the lowest binding interaction energy was selected. From our models, PPO has a slightly smaller binding pocket than POD. Therefore, the number of binding amino acid residues was observed. All the residues with less than 3 Å distances to epicatechin are represented in Figure 3, including His87, Phe113, Asn240, His243, Lys244, Gly257, Phe259, Ala262, Phe268 for grape polyphenol oxidase and Arg37, Ala69, Asn71, Leu130, Pro131, Asn132, Ala133, Ser171 for ascorbate peroxidase of grape. For comparison, the energies obtained from the docking of each ligand are listed in Table 2. The more negative interaction energies exhibit the more favorable binding. The prediction of interaction energy with the substrates and inhibitors of grape peroxidase are generally lower than that of grape polyphenol oxidase. The substrates with high affinity were epicatechin and catechin with −45.63 and −44.75 kcal/mol for peroxidase and −42.99 and −45.55 kcal/mol for polyphenol oxidase, respectively. Other complexes did not show a difference in binding affinity according to the interaction energy range from −25.91 to −35.46 kcal/mol for peroxidase and −23.93 to −53.55 kcal/mol for polyphenol oxidase. The selected ligands frequently form hydrogen bonds with Gly257 of grape polyphenol oxidase and Arg37 of grape ascorbate peroxidase. Hydrogen bond interactions were determined using the following criteria: (i) The distance between proton donor (D) and acceptor (A) atoms ≤ 3.5 Å and (ii) the D-H.A angle = 120°. Similary, Tatoli, *et al.* had reported the strong hydrogen bonding between the Arg38 side chain and peroxy-complex of recombinant horseradish peroxidase, which is one of the most studied enzymes among the heme peroxidases for its importance in modern enzymology [14]. A commonly accepted mechanism for peroxidases proposed many years ago by Poulos-Kraut [15] has also reported the importance of the highly conserved His42 and Arg38 residues in the stepwise acid-base catalysis.

### 2.3. Specificity of Inhibitors for PPO and POD: Theoretical and Experimental Comparison

From experimental studies, various potent inhibitors for grape polyphenol oxidase were ascorbic acid, cysteine, and sodium metabissulfite [16], whereas cysteine inhibited polyphenol oxidase activity in mango puree [17] and was effective in preventing the browning of apple juice [18,19]. However, cysteine produces an undesirable order, limiting its use in food processing. The aromatic carboxylic acids (benzoic and cinnamic acid) were inhibitors, due to their structural similarity with phenolic substrates [18]. In order to study the binding mode of the inhibitors, benzoic acid and its analogs shown to control enzymatic browning [20] were chosen for the investigation. The calculated docking interaction energy of benzoic compounds showed high affinity to grape ascorbate peroxidase and polyphenol oxidase (Table 2). Ferrer and coworker reported that 2,3-dihydroxybenzoic acid showed no inhibitory effect whereas 2,4-dihydroxybenzoic acid was a strong polyphenol oxidase inhibitor [21]. From our docking study, the inhibitor 3,4,5-trihydroxybenzoic acid has high affinity with both enzymes. The series of monohydroxybenzoic acids (*m-*, *o-*, *p-*hydroxybenzoic acid) have high affinities with grape polyphenol oxidase with lower negative interaction energy values than those with peroxidase. Other compounds, including 2,3-dihydroxybenzoic acid, 3,4-dihydroxybenzoic acid, *o-*hydroxybenzoic acid, and *m-*hydroxybenzoic acid, can be used as common inhibitors for both enzymes.

## 3. Experimental Section

### 3.1. Three Dimensional Structure Modeling

The sequence of grape ascorbate peroxidase was obtained from Entrez Protein of NCBI (accession number ABX79340). The BLAST search [12] was used to identify homologous proteins against the current Protein Data Bank (PDB: http://www.rcsb.org). In order to find a template for homology modeling, we used the BLAST Search (DS-server) from Discovery studio 1.7 program. We used the crystal structure of pea cytosolic ascorbate peroxidase (PDB ID:1APX) [22] as the template to build the 3D structure of grape ascorbate peroxidase. Several initial models were constructed, using Modeler module [23] in Discovery studio 1.7, and the one with highest score of the Profiles-3D was retained. To refine the initial homology model, the CHARMm force field was employed and the following energy minimization procedures were processed. The minimization was carried out while the heme was constrained and other atoms were allowed to relax. Minimization procedure was used with the steepest descent method for 1000 steps. Finally, the quality of residue backbone conformation was checked by PROCHECK.

### 3.2. Docking Study

The established grape ascorbate peroxidase homology model or the x-ray structure of grape polyphenol oxidase (PDB ID:2P3X) was used as the receptor. To prepare the crystal structure of polyphenol oxidase, the protein was purified from Grenache grape berries by using traditional methods and crystallized with ammonium acetate by the hanging-drop vapor diffusion method. The structure was obtained at 2.2 Å resolution. Energy minimization was performed by using 1,000 steps of steepest descent method. Schematic representations of the ligand for each of enzymes used in this study are shown in Table 2. The 3D structures of ligands were sketched and optimized with the AM1 method. A CHARMm-based docking program CDOCKER algorithm [24] was employed to find the potential binding mode between both enzymes and the phenolic compound ligand. The active site pocket of the receptor was found automatically by the Discovery Studio1.7. The site sphere radius of 14 Å of grape peroxidase and 7 Å of grape polyphenol oxidase were set to assign the entire ligand binding pocket. In CDOCKER, random ligand conformations are generated through molecular dynamics, and a variable number of rigid-body rotations/translations are applied to each conformation to generate the initial ligand poses. The conformations were further re ned by grid-based simulated annealing in the receptor active site, which makes the results accurate. The CDOCKER interaction energy between the substrates/inhibitors to enzymes was nally computed. The complex structure with the lowest interaction energy was used for comparison.

## 4. Conclusions

The three dimensional model of peroxidase from grape (*Vitis vinifera*) was constructed from 1APX with high identity (80%) and high resolution (2.20 Å). The Ramachandran plot of phi and psi distribution of grape ascorbate peroxidase homology model was well within a reliable model with 95.6% residues in most favored regions and 4.4% residues in additional allowed regions. Evaluation score from Verify 3D analysis suggested that minimized grape ascorbate peroxidase homology model was a sufficient model for further enzyme-substrate docking study.

The docking calculations reveal that phenolic and benzoic compounds bind in the active site of grape ascorbate peroxidase and polyphenol oxidase with various degrees of affinity. The prediction interaction energy of grape ascorbate peroxidase is generally lower than that of grape polyphenol oxidase. Substrates with high affinity to both enzymes are epicatechin and catechin. The calculated docking interaction energy of 3,4,5-trihydroxybenzoic acid showed the high affinity, suggesting specificity and potential use as a common inhibitor to grape ascorbate peroxidase and polyphenol oxidase.

## Figures and Tables

**Table 1:** Quality of structures checked by PROCHECK for model and template.

PROCHECK	Ramachandran Plot Quality (%)			
	Core	Allowed	General	Disallowed
Model	95.6	4.40	0	0
Template	93.7	5.8	0	0.5

**Table 2:** Experimental predicted interaction of phenolic and benzoic acid compounds with grape ascorbate peroxidase and polyphenol oxidase.

Substrate	Structure	ABX (POD)				2P3X (PPO)			
		Experimental Value [10] K_m_(×10^−3^ M)	Interaction Energy (kcal/mol)	No. of Hydrogen Bonding	Residue in hydrogen Bonding	Relative Activity [6]	Interaction Energy (kcal/mol)	No. of Hydrogen Bonding	Residue in Hydrogen Bonding
**Substrates**									
4MC	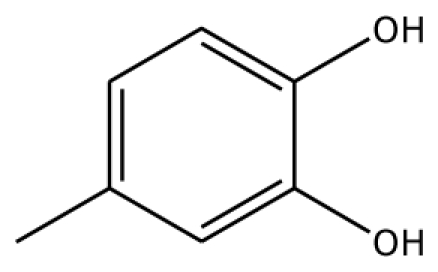	22.0	−28.23	1	Arg37	100	−41.85	1	His239
GAC	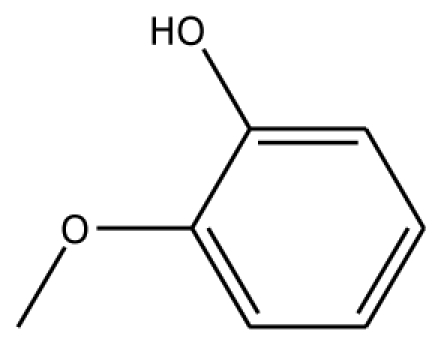	32.2	−28.49	2	Arg37		−23.93	0	
PGL	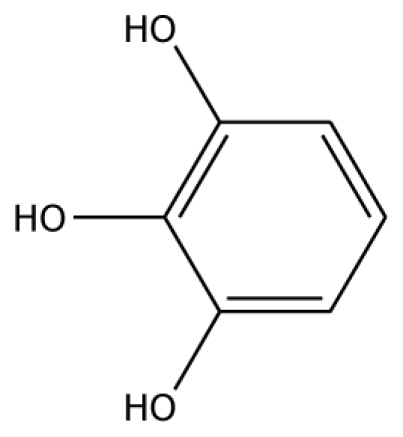	32.2	−30.45	2	Arg37	78.1	−28.78	0	
3,4-DHPA	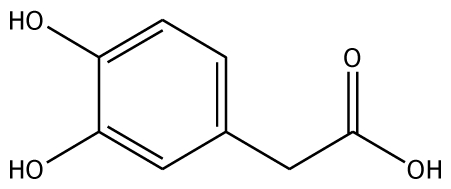	na	−35.46	2	Trp40 Arg170	na	−53.55	2	His239 Gly257
CN	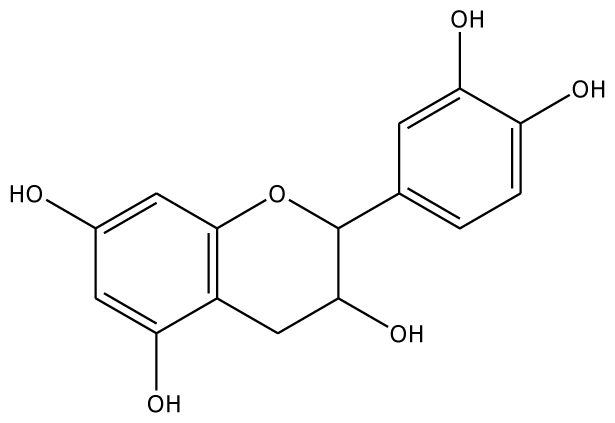	5.2	44.75	2	Arg37 Glu68	na	−45.55	2	Asn240 Gly257
EPC	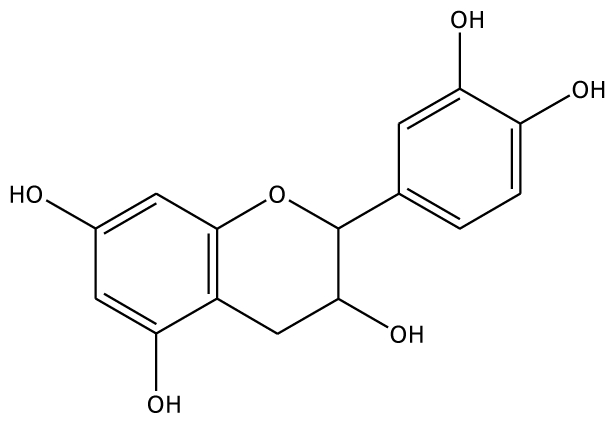	5.2	−45.63	2	Arg37 Glu68	93.1	−42.99	1	Asn240

**Inhibitors**									
2,3-DHBA	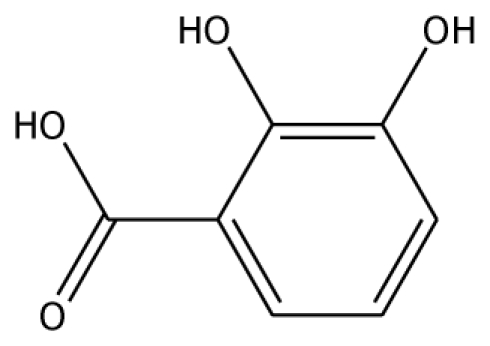	na	−32.15	1	Pro131	na	37.37	1	Gly257
3,4-DHBA	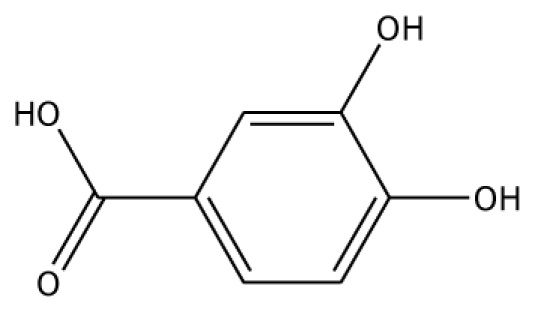	na	−31.38	1	Arg170	na	−44.71	1	His239
3,4,5-THBA	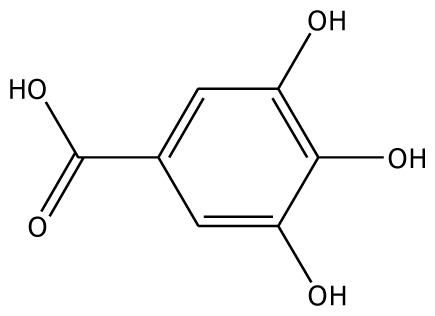	na	34.76	1	Arg37	na	−43.01	4	His239His243 Gly257 Asn258
*o*-HBA	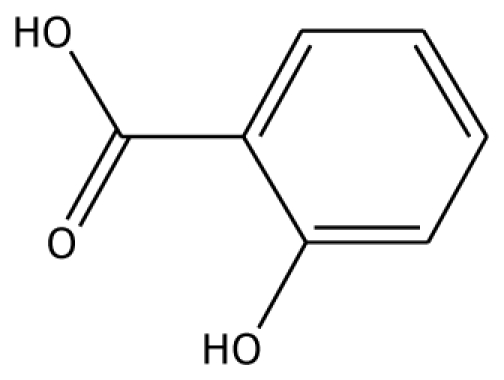	na	−29.14	1	Arg37	na	−33.99	1	His239
*m*-HBA	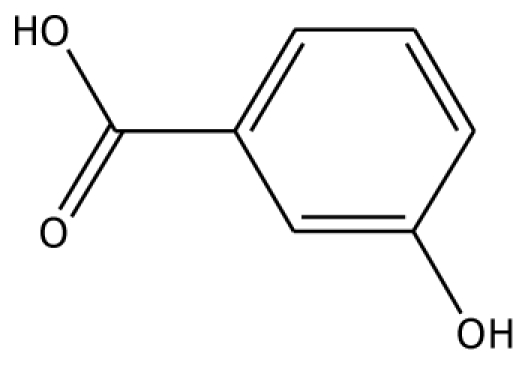	na	−29.17	0		na	−39.04	1	Gly257
*p*-HBA	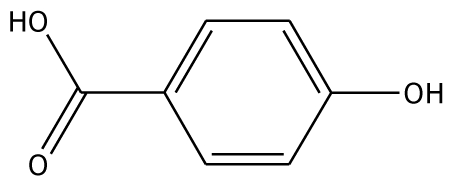	na	−26.23	1	Trp40	na	−36.68	2	Glu235 Gly257

Abbreviations: 4MC, 4-methylcatechol; PGL, pyrogallol; GAC, guaiacol; 3,4-DHPA, 3,4-dihydroxyphenylacetic acid; CN, catechin; EPC, epicatechin; 2,3-DHBA, 2,3-dihydroxybenzoic acid; 3,4-DHBA, 3,4-dihydroxybenzoic acid;3,4,5-THBA, 3,4,5-trihydroxybenzoic acid; *o*-HBA, *o*-hydroxybenzoic acid; *m*-HBA, *m*-hydroxybenzoic acid; *p*-HBA, *p*-hydroxybenzoic acid.
